# Milling Chatter Control in Low Immersion Condition with an Active Electromagnetic Tool Holder System

**DOI:** 10.3390/mi16030257

**Published:** 2025-02-25

**Authors:** Chen Wang, Haifeng Ma, Jie Chen, Zhen Zhang, Qinghua Song, Zhanqiang Liu

**Affiliations:** 1 School of Mechanical Engineering, Shandong University, Jinan 250061, China; 202214327@mail.sdu.edu.cn (C.W.); chen_jay@mail.sdu.edu.cn (J.C.); ssinghua@sdu.edu.cn (Q.S.); melius@sdu.edu.cn (Z.L.); 2 State Key Laboratory of Advanced Equipment and Technology for Metal Forming, Shandong University, Jinan 250061, China; 3 Key Laboratory of High Efficiency and Clean Mechanical Manufacture of Ministry of Education, Jinan 250061, China; 4 Shandong Key Laboratory of High Performance Tools and System, Jinan 250061, China; 5 Shandong Provincial Key Laboratory of Computer Networks, Shandong Fundamental Research Center for Computer Science, Jinan 250014, China; zhenzhang@sdas.org; 6 Key Laboratory of Computing Power Network and Information Security, Ministry of Education, Shandong Computer Science Center (National Supercomputer Center in Jinan), Qilu University of Technology (Shandong Academy of Sciences), Jinan 250014, China; 7 School of Mechanical, Electrical & Information Engineering, Shandong University, Weihai 264209, China

**Keywords:** milling chatter control, low immersion milling, electromagnetic actuator, weak rigidity of thin-walled workpiece and tool

## Abstract

Chatter commonly emerges during milling procedures, resulting in an array of problems such as defective workpiece surface and diminished machining efficiency. To control chatter, an active electromagnetic tool holder system is proposed, including the active structure with an electromagnetic actuator installed at the tool holder position and a time-delay output feedback chatter control method for low immersion milling. More specifically, a noncontact two-degree-of-freedom active magnetic bearing (AMB) actuator is developed and integrated with displacement sensors at the tool holder position, making the actuator and sensors closer to the cutting point. Under low immersion milling conditions, both the thin-walled workpieces and tool flexibility are considered in the controller design, as well as practical physical limitations including the bandwidth of the power amplifier and the output current constraints of the actuator. Numerical simulation and experiments under low immersion milling conditions are carried out. The results demonstrate that the proposed active electromagnetic tool holder system exhibits good control consequences on the chatter of thin-walled workpieces and tools under low immersion milling.

## 1. Introduction

The advancement of manufacturing technology has led to increased demands for high efficiency and precision in processing. In traditional machining systems, the tool structure has low rigidity, and it is easy to produce chatter dominated by the tool mode during the cutting process. Additionally, the inherent low rigidity of thin-walled workpieces further exacerbates the occurrence of chatter, which is primarily governed by the workpiece’s modal behavior. This phenomenon significantly undermines the surface quality and processing efficiency [1,2]. Consequently, numerous passive and active methods have been developed and refined to suppress vibration [3,4]. This paper focuses on regenerative chatter.

Passive suppression strategies involve the utilization of customized tool structures, such as variable pitch tools, variable helix tools, and serrated tools [5,6,7]. These tools modify the delay between the tool edges by adjusting the irregularities in their geometric shapes, thereby disrupting the regenerative process and achieving chatter suppression. In addition, there are traditional passive suppression methods. One option of the traditional passive suppression strategy is to improve the system damping and absorb the chatter energy by using impact dampers [8], tuned mass dampers [9], vibration absorbers [10], and so on. Another option is based on increasing the stiffness of the machining system, which is achieved by improving the strength of the workpiece through appropriate clamping elements or auxiliary supports, such as the use of ice supports [11] on the workpiece, magnetorheological damping fixtures [12], and other new types of fixtures, ultimately aiming to achieve the purpose of suppressing chattering. The use of these passive control methods can effectively expand the stable chatter region. Nevertheless, the adaptability of these methods to various cutting conditions or workpieces needs to be further explored.

The active chatter control method utilizes sensor measurements as feedback signals. The control force applied to the machining system is calculated and implemented in real-time through a control algorithm, thereby effective chatter control during the machining process can be achieved. The modeling of tools and machining systems [13,14] enables a deeper understanding of the factors affecting machining stability and is the basis for active control. Common active actuators include piezoelectric actuators, active magnetic bearings (AMBs), and electrostrictive stack actuators [15,16,17,18,19,20,21,22,23,24,25,26,27,28,29,30,31]. For milling processes, active control systems can be categorized into three types based on the installation locations of sensors and actuators: active spindle system, active tool holder system, and active workpiece system. The active spindle system is achieved by modifying the traditional spindle and integrating various types of actuators into the spindle system. Wu et al. [15] developed a five-degree-of-freedom electromagnetic levitation spindle supported fully by AMBs. Wan et al. [16,17] and Li et al. [18] utilized electromagnetic spindles supported by both AMB and mechanical bearings. Monnin et al. [19,20] distributed four sets of piezoelectric actuators radially uniformly on the outer ring of the front section bearing of the spindle. Experiments demonstrated that these methods can enhance the critical stable depth of cut in milling operations. Nevertheless, this approach to spindle transformation often necessitates a significant integration space, and the location of force application and sensors is considerably distant from the cutting point [21]. The widely reported active tool holder system is achieved through the utilization of customized tool holders and additional mechanical bearings [22,23,24,25,26]. The control force must be applied indirectly to the rotating tool or tool holder via the additional bearing. Due to the applied radial force and the preload required for mounting, the extra heat generated in the bearing cannot be overlooked, as it may adversely affect the bearing’s temperature and operational lifespan. Furthermore, the piezoelectric actuator exhibits nonlinear hysteresis, which can impact the control efficiency in practical applications. For certain thin-walled workpieces with high flexibility, the control force is usually applied directly to the workpiece by a piezoelectric sheet or piezoelectric actuator [27,28,29,30]. However, it is necessary to consider the influence of the actuator position on the control effect. When facing the machining of complex thin-walled parts, there is a certain lack of control force in the fixed position.

With the deepening of the understanding of vibration, various controllers such as sliding mode controllers [17], robust controllers [30,31,32], fuzzy-PID controllers [33], model predictive controllers [34,35], optimal controllers [26], and other novel control strategy [36] have been increasingly implemented in active control [37]. These algorithms significantly improve the stability of cutting depth and effectively suppress chatter. Low immersion milling is a prevalent machining condition for thin-walled workpieces, and research on these machining conditions is generally limited to stability analysis and prediction [38,39,40]. Researchers considering active control often overlook the low immersion milling conditions and utilize milling force coefficients under full immersion milling [25,31,41]. Only a limited number of algorithms employ high-order approximations to estimate nonsmooth milling forces under low immersion conditions. Wu et al. [15] analyzed milling chatter control by employing a lower (respectively, upper) autonomous model with time-varying coefficients as uncertain factors. Huang et al. [42] and Chen et al. [43] developed an adaptive controller for the second-order approximate milling force coefficient. Du et al. [32] proposed a robust combined time-delay control method regarding the nonsmooth milling coefficient and dynamic characteristics as parameter disturbances. Cao et al. [44] introduced a first-order segmented model that approximated the low immersion milling system as a time-invariant submodel with two cycles of switching. The designed controller can effectively suppress vibration at low immersion ratios. However, a complex approximation process may increase the order of the controller, presenting challenges for its design. Alternatively, chatter may originate from either the tool or the workpiece. Existing studies on low immersion milling conditions primarily focus on either the chatter of thin-walled workpieces or the weak rigidity of the cutting tool, without investigating the coupled chatter effects of both components. Moreover, in practical applications, the physical limitations of the actuator should always be fulfilled in active chatter control, including bandwidth constraints and output current constraints. The physical limitations are not addressed in [37,42,43]. Although the output current constraints are discussed in [15], the bandwidth of the power amplifier is overlooked. In the active control of low immersion milling, there remains a lack of research that takes both the bandwidth and current constraints into account.

To address the aforementioned issues, a novel active control system is proposed in this paper for milling chatter, including an active structure with the electromagnetic actuator installed at the tool holder position and a time-delay output feedback chatter control method for low immersion milling. The active structure positions the noncontact active magnetic bearing closer to the cutting point, thereby enhancing its ability to perceive and control vibration. The controller is designed specifically for low immersion milling, taking into account the flexibility of the workpiece and tool, and effectively mitigating chatter in both components. Furthermore, practical physical constraints, such as the bandwidth of the power amplifier and the output current constraints of the actuator, are incorporated into the controller design. To validate the effectiveness of the proposed system, simulations and experiments are conducted under conditions of low immersion milling.

The remainder of this paper is organized as follows: The developed active tool holder structure along with its operational principles are presented in Section 2. Building upon this foundation, a theoretical model that accounts for the flexibility of thin-walled workpieces and tools is established. In Section 3, a time-delay output feedback controller for low immersion milling conditions is designed. Numerical simulations and machining experiments under different conditions are carried out in Section 4 and Section 5, respectively. Finally, Section 6 provides the conclusions drawn from this study.

## 2. System Modeling

In this section, an active tool holder structure with an electromagnetic actuator and sensors installed at the tool holder position is designed. The foundational principles of this structure, along with its potential applications, are discussed. Furthermore, the dynamic model that accounts for the flexibility of thin-walled workpieces and tools is built based on the structure.

### 2.1. Design of the Active Tool Holder Structure

**Figure 1:**
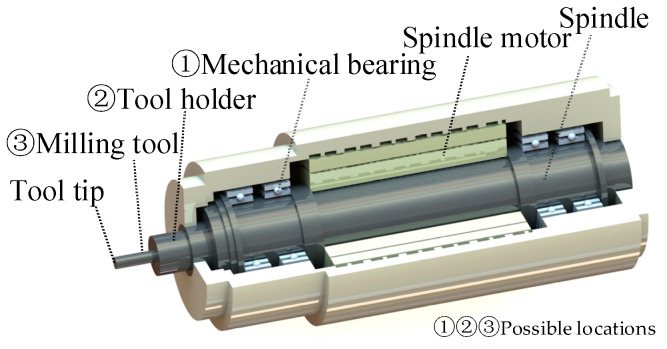
Possible locations for the actuator integration.

In numerous milling machining applications, active control structures are incorporated into rotating systems. The potential integration positions are illustrated in Figure 1.

The spindle system primarily comprises the spindle motor, spindle, supporting mechanical bearings, etc. A significant advantage of directly integrating sensors and actuators into the spindle system is the substantial integration space it offers. The active spindle structure, which utilizes AMBs as the primary component, as referenced in studies [16,17,18,19], involves replacing one or all mechanical bearings (designated as position ① in Figure 1) with AMBs. These AMBs provide the necessary bearing capacity and facilitate vibration control when combined with displacement sensors. Nevertheless, this structural modification necessitates alterations to the spindle, which is positioned at a distance from the cutting point. Consequently, the effectiveness of vibration monitoring and control diminishes as the signal is transmitted from the tool to the spindle. This attenuation results in limitations in the system’s ability to respond to and adjust for vibration. Furthermore, altering the front bearing may adversely affect the spindle’s performance, leading to increased economic costs and challenges in subsequent maintenance.

The tool holder system represents a distinct subsystem within the rotating system, encompassing both the cutting tool and the tool holder. Cutting tools, such as milling cutters and drilling cutters, are affixed to the tool holder through a mechanical interface. One potential method for mounting the actuator involves positioning it on the tools (position ③ in Figure 1). This location is advantageous due to its proximity to the cutting point. However, it presents significant limitations in terms of installation space and must also take into account high mechanical, the thermal loads and cutting fluid. Another alternative strategy is to modify the tool holder (position ② in Figure 1). The active tool holder structure that employs piezoelectric actuators, as referenced in studies [20,21,22,23,24,25,26,27], includes an additional mechanical bearing as an auxiliary component. In this configuration, the control force is transmitted to the bearing, allowing the force to indirectly affect the tool holder. Nonetheless, this approach may negatively impact the temperature and operational lifespan of the auxiliary bearing. Meanwhile, the inherent nonlinear hysteresis of the piezoelectric actuator can diminish efficiency in practical applications.

**Figure 2:**
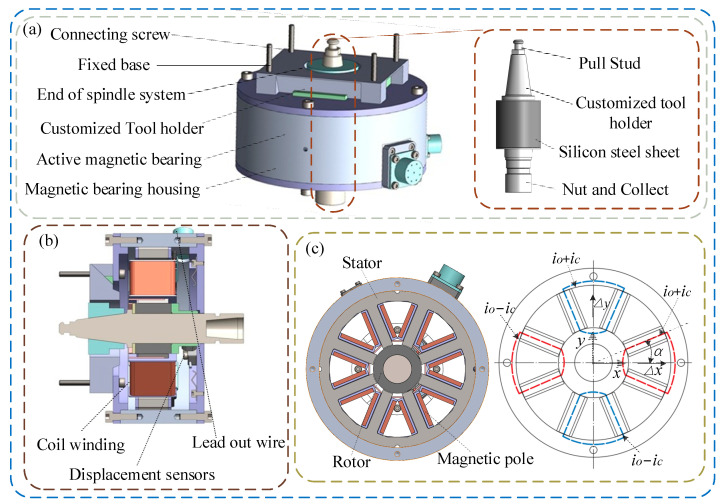
Designed active tool holder control structure: (**a**) Structure installation diagram. (**b**) Section diagram. (**c**) Schematic diagram of active magnetic bearing.

In response to these challenges, an active tool holder structure that utilizes AMB as the actuator is proposed. This design allows the generated force to exert a direct influence on the tool holder. Moreover, the AMB near the end of the rotating system is closer to the cutting point, which enhances its sensitivity and control capabilities concerning vibration. The designed active tool holder structure is shown in Figure 2, including the AMB as stator, and the customized tool holder as rotor, as well as the eddy current displacement sensors for measuring the vibration signal.

The AMB is characterized by its noncontact, nonlubricated, and nonwearing properties. By manipulating the bias current alongside a control current, the AMB can generate a controllable electromagnetic field force, which facilitates the suppression of vibration in the rotating tool. The designed AMB is an eight-stage radial active magnetic bearing with two degrees of freedom, calculated according to the actual milling machine size. The primary parameters of the AMB are delineated in Table 1. To mitigate potential damage, the outer ring of the tool holder is reinforced with silicon steel sheets.

The stator features eight symmetrically distributed magnetic poles, with the coils of two adjacent magnetic poles connected in pairs. When current flows through the coils, it generates a magnetic flux loop and electromagnetic force, as illustrated in Figure 2. Two symmetric electromagnetic pairs are working together in the *x* and *y* directions, respectively, driven by the control current *i_c_* and bias current *i*_0_. One pole pair is controlled by *i*_0_ + *i_c_*, and another electromagnetic pair is controlled by *i*_0_ − *i_c_*. The control force imposed by the AMB in each direction can be indicated as in Equation (1).

(1) $$F_{a}\left(t\right)=F_{+}-F_{-}=\frac{1}{4}\mu_{0}A_{n}N^{2}\left(\frac{{\left(i_{0}+i_{c}\right)}^{2}}{{\left(c_{0}-z_{s}\right)}^{2}}-\frac{{\left(i_{0}-i_{c}\right)}^{2}}{{\left(c_{0}+z_{s}\right)}^{2}}\right)\cos\alpha$$

where *μ*_0_ is the vacuum permeability, *A_n_* is the magnetic circuit’s cross-sectional area, *z_s_* is the rotor displacement signals measured by integrated eddy current displacement sensors. During the milling process, the displacement variation Δ*z_s_* is much smaller than the air gap *c*_0_ value. *F_a_* can be simplified and linearized as

(2) $$F_{a}(t)=k_{i}i_{c}+k_{s}z_{s}$$

with $k_{i}=\frac{\mu_{0}AN^{2}i_{0}}{{c_{0}}^{2}}cos\alpha ,{\;k}_{s}=-\frac{\mu_{0}AN^{2}{i_{0}}^{2}}{{c_{0}}^{3}}cos\alpha$, and *k_i_*, *k_s_* are denoted as current stiffness coefficient and displacement stiffness coefficient, respectively.

The vibration responses of the tool holder are measured by sensors in both *x* and *y* directions, preprocessed, and fed back to the controller during the milling process. These signals are then preprocessed and sent back to the controller. Through the power amplifier, the control voltage produced by the controller is converted into current. Finally, the control force is generated to suppress chatter. The maximum output current of the power amplifier is 8 A, and the input voltage of 1 V corresponds to the output current of 1 A. The effective operating bandwidth of the power amplifier is within 1500 Hz.

### 2.2. Model of the Coupled System Under Low Immersion Milling

**Figure 3:**
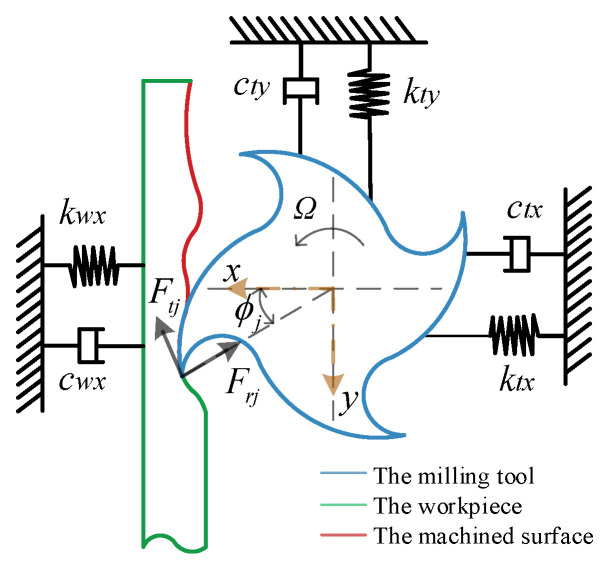
Model of milling system.

During the milling process, both the cutting force and thickness change as the tool rotates, resulting in continuous vibrations in both the tool and the workpiece. These vibrations subsequently influence the cutting thickness. To capture the dynamic characteristics of the tool and workpiece systems, the tool system is modeled as a two-degree-of-freedom system, with its *x* and *y* axes oriented perpendicularly to each other. Conversely, the flexible thin-walled workpiece is considered a one-degree-of-freedom system. This two-dimensional model of milling is illustrated in Figure 3.

The dynamic displacement is generated by the forces acting in the two directions, respectively. During milling, the cutting thickness *h*(*ϕ_j_*) associated with the regenerative effect is divided into two parts: the static cutting thickness *h_s_*, which is attributed to the rigid body motion of the tool, and the dynamic cutting thickness *h_d_*, which is a result of the tool’s vibrations during the current and previous tooth engagements.

(3) $$\begin{array}{l} h_{s}=f_{z}\sin\phi_{j},h_{d}=-x\sin\phi_{j}-y\cos\phi_{j}\\ h\left(\phi_{j}\right)=fz\sin\phi_{j}+\Delta x\sin\phi_{j}+\Delta y\cos\phi_{j}]g(\phi_{j}) \end{array}$$

where *f_z_* denotes the feed per tooth, and *ϕ_j_* represents the instantaneous angular position of tooth *j*, measured in a clockwise direction from the normal *y*-axis. Δ*x = x* − *x*_0_ and Δ*y* = *y* − *y*_0_ denote the dynamic displacement of the tool structure during the current and previous tooth intervals.

The time delay *τ = 2π/NΩ* corresponds to the cutter’s cutting cycle, where the spindle speed is denoted as *Ω*. The milling is decomposed into tangential and radial directions when modeling. The tangential cutting force *F_tj_*(*t*) and radial cutting force *F_rj_*(*t*) exerted on tooth *j* are in direct proportion to the axial cutting depth b and the chip thickness *h*(*ϕ_j_*).

(4) $$F_{tj}=K_{t}bh\left(\phi_{j}\right),\;F_{rj}=K_{r}F_{tj}$$

where *K_t_* represents the tangential milling force coefficient, and *K_r_* represents radial milling force coefficient. The ratio *K_n_* is defined as *K_n_ = K_t_/K_r_*. Based on the chip thickness, the milling force acting on the entire milling tool can be characterized. The milling force can be partitioned into two components: the static milling force *F_s_* and the dynamic milling force *F_d_*. Both of *F_s_* and *F_d_.* are periodic.

(5) $$\begin{array}{l} F_{t}=F_{S}+F_{d}\\ F_{S}=\frac{1}{2}bf_{z}K_{t}H_{S}\left(t\right),F_{d}=\frac{1}{2}bH_{d}\left(t\right)\Delta Z\left(t\right) \end{array}$$

where Δ*Z*(*t*) = [Δ*x* Δ*y*] ^T^ represents the dynamic chip thickness. As we are only interested in the part that results in chatter, the static component of the milling force can be ignored. Based on the above equation, the cutting force change matrix is obtained.

(6) $$H_{d}(t)=K_{t}\sum_{j=0}^{N-1}g\left(\phi_{j}\right)\left[\begin{array}{cc} -\sin2\phi_{j}-K_{n}(1-\cos2\phi_{j}) & -K_{n}\sin2\phi -(\cos2\phi_{j}+1)\\ -K_{n}\sin2\phi_{j}+(1-\cos2\phi_{j}) & \sin2\phi -K_{n}(\cos2\phi_{j}+1) \end{array}\right]$$

where *g*(*ϕ_j_*) is a unit step function. *ϕ_en_* and *ϕ_ex_* are the starting and exiting immersion angles between the tool and the entry point, respectively.

(7) $$g\left(\phi_{j}\right)=\left\{\begin{array}{c} 1,\phi_{en}\leq \phi_{j}\leq \phi_{ex}\\ 0,\phi_{j}<\phi_{en}or\phi_{j}>\phi_{ex} \end{array}\right.$$

Meanwhile, in the up milling, *ϕ_en_* = 0, *ϕ_ex_* = arcos (1 − 2*a/D*). If down milling is performed, *ϕ_en_* = arcos (2*a/D* − 1), *ϕ_ex_* = π. Within the coefficient matrix, the ratio of the radial cutting depth to the milling tool diameter is denoted as *a/D*, where *a* ∈ [0, *D*]. When *a/D* = 1, this milling mode is referred to as the full radial immersion milling. In this particular scenario, the entire milling tool is submerged in the workpiece material. As *a* decreases gradually, the ratio *a/D* also decreases, ultimately reaching the state of low radial immersion milling. In the stability analysis and predictive research for low immersion milling conditions, a high-order approximation of the Fourier series can be employed to calculate an accurate cutting force coefficient matrix.

However, in active control scenarios, it is not essential to prioritize model accuracy excessively; instead, practical applications demand the focus be centered on effectively managing the machining process. The purpose of the zero-order approximation is to streamline the system model. By transforming the time-varying system into a time-invariant one, which sufficiently meets the requirements.

The cutting force variation matrix *H*(*t*) exhibits periodicity at all the tooth passing frequencies *ω = NΩ*, which is consistent with the cutting force. Equation (8) can be obtained by Fourier series expansion of Equation (7).

(8) $$H(t)=\frac{K_{t}}{2}\frac{1}{\tau }\int_{0}^{\tau }H_{d}(t)dt=K_{t}\frac{N}{4\pi }\left[\begin{array}{cc} h_{11} & h_{12}\\ h_{21} & h_{22} \end{array}\right]\left|\begin{array}{c} \phi_{ex}\\ \phi_{en} \end{array}\right.$$

where

$$\begin{array}{l} h_{11}=\frac{1}{2}\left[\cos2\phi -2K_{n}\phi +K_{n}\sin2\phi \right]\left|\begin{array}{c} \phi_{ex}\\ \phi_{en} \end{array}\right.,h_{12}=\frac{1}{2}\left[-\sin2\phi -2\phi +K_{n}\cos\phi 2\phi \right]\left|\begin{array}{c} \phi_{ex}\\ \phi_{en} \end{array}\right.\\ h_{21}=\frac{1}{2}\left[-\sin2\phi +2\phi +K_{n}\cos2\phi \right]\left|\begin{array}{c} \phi_{ex}\\ \phi_{en} \end{array}\right.,h_{22}=\frac{1}{2}\left[-\cos2\phi -2K_{n}\phi -K_{n}\sin2\phi \right]\left|\begin{array}{c} \phi_{ex}\\ \phi_{en} \end{array}\right. \end{array}$$

After applying zero-order approximate averaging to the above equation, Equation (9) is obtained.

(9) $$H_{0}(t)=K_{t}\frac{N}{8\pi }\left[\begin{array}{cc} \cos2\phi -2K_{n}\phi +K_{n}\sin2\phi & -\sin2\phi -2\phi +K_{n}\cos2\phi \\ -s\mathrm{in}2\phi +2\phi +K_{n}\cos2\phi & -\cos2\phi -2K_{n}\phi -K_{n}\sin2\phi \end{array}\right]\left|\begin{array}{c} \phi_{ex}\\ \phi_{en} \end{array}\right.$$

**Figure 4:**
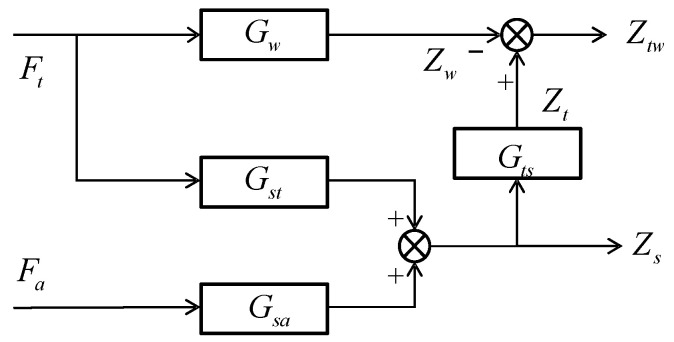
System transfer function.

Chatter can originate from either the tool or the workpiece. A coupled milling system model, which takes into account thin-walled workpieces and weakly rigid tools, as depicted in Figure 4, has been established. *F_t_* and *F_a_* represent the total milling force caused by feed motion during the milling process and the controlled force provided by the active magnetic bearing, respectively.

The displacement at the tooltip is denoted as *Z_t_*, while *Z_w_* represents the displacement of the workpiece. Additionally, *Z_tw_*(*t*) = *Z_t_*(*t*) − *Z_w_*(*t*) refers to the relative displacement between the tool and the workpiece. For this coupled system model, the regenerative effect model is constructed by considering the relative time-delay displacement Δ*Z_tw_*(*t*) = Δ*Z_t_*(*t*) − *Z_w_*(*t*). *Z_s_* is the detected displacement signal of the actuator position on the tool holder. In this system, it serves as the feedback for realizing the coupled chatter control.

Figure 4 shows the corresponding relationship between the transfer functions. *G_w_*(*s*) are denoted as the transfer function from the milling force to the workpiece, while *G_st_*(*s*) are designated as from the milling force to the measured position of the spindle system. The transfer function of the controlled force generated by the actuator to the measured position is embodied in *G_sa_*(*s*). Meanwhile, *G_ts_*(*s*) represents the transfer function from the measured position to the tooltip. In the frequency domain, the milling system can be described by Equation (10).

(10) $$\begin{array}{l} Z_{w}(s)=G_{w}(s)F_{t}(s),\\ Z_{s}(s)=\left[\begin{array}{cc} G_{st}(s) & G_{sa}(s) \end{array}\right],\\ Z_{t}\left(s\right)=G_{ts}\left(s\right)Z_{s}\left(s\right)=G_{ts}\left(s\right)\left[\begin{array}{cc} G_{st}\left(s\right) & G_{sa}\left(s\right) \end{array}\right]{\left[\begin{array}{cc} F_{t}\left(s\right) & F_{a}\left(s\right) \end{array}\right]}^{T} \end{array}$$

After collation, the system model of Equation (11) is obtained.

(11) $$\left[\begin{array}{c} Z_{tw}(s)\\ Z_{s}(s) \end{array}\right]=\left[\begin{array}{cc} G_{ts}(s)G_{st}(s)-G_{w}(s) & G_{ts}(s)G_{sa}(s)\\ G_{st}(s) & G_{sa}(s) \end{array}\right]\left[\begin{array}{c} F_{t}\left(s\right)\\ F_{a}\left(s\right) \end{array}\right]$$

For the sake of facilitating the subsequent analysis, Equation (11) can be represented in the form of a state space equation, which is shown as Equation (12).

(12) $$\begin{array}{l} \dot{x}(t)=Ax(t)+BF(t)\\ z(t)=Cx(t) \end{array}$$

where ${F(t)=\left[\begin{array}{cc} F_{t}\left(t\right) & F_{a}\left(t\right) \end{array}\right]}^{T}$ is the system input, $z(t)={\left[\begin{array}{cc} C_{tw} & C_{s} \end{array}\right]}^{T}$ is the system output, $B=\left[\begin{array}{cc} B_{t} & B_{a} \end{array}\right]$, $C={\left[\begin{array}{cc} C_{tw} & C_{s} \end{array}\right]}^{T}$. Equation (2) can be substituted into the above equation and simplified to obtain Equation (13).

(13) $$\begin{array}{l} \dot{x}(t)=\left(A+B_{t}bH_{0}C_{tw}+B_{a}k_{s}C_{s}\right)x(t)-B_{t}bH_{0}C_{tw}x(t-\tau )+B_{a}k_{i}i_{c}\\ z(t)=Cx(t) \end{array}$$

## 3. Controller Design

In this section, a time-delay output feedback chatter controller for low immersion milling is presented, which takes into account the coupled system Equation (13), as well as practical physical limitations, including the bandwidth of power amplifier Equation (15) and the output current constraints of actuator Equation (24) in the controller design. The coupled dynamic model augmented by weighting functions Equation (18) is used for the output feedback controller synthesis. To solve the synthesis problem, a set of linear matrix inequalities (LMIs) and the Schur complement lemma are derived and adopted.

### 3.1. Augmented Model

The objective of the controller is to achieve the suppression of the vibration during the machining process. The performance index focused on minimizing the coupled system displacement in the desired specific frequency region. In practical applications, due to the bandwidth limitation of the power amplifier output, it is necessary to consider the bandwidth of the power amplifier and output current constraints of the actuator. As for the bandwidth of the power amplifier, the frequency-dependent weighting function is employed.

**Figure 5:**
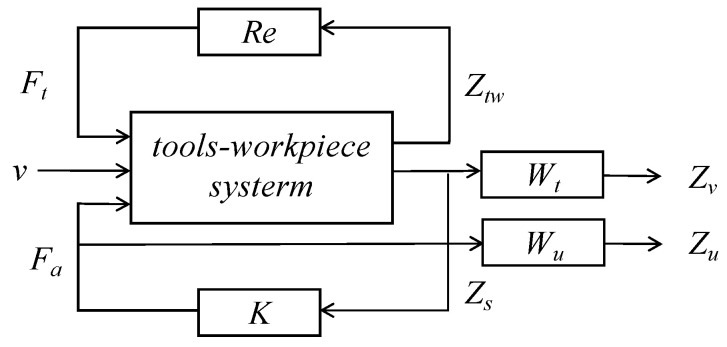
System control block diagram.

Set performance variables *W_v_*(*s*) = *diag*{*w_v_*(*s*), *w_v_*(*s*)}, and control variables *W_u_*(*s*) = *diag*{*w_u_*(*s*), *w_u_*(*s*)}, as shown in Figure 5.

(14) $$w_{v}(s)=\frac{z_{v}}{z_{s}}=K_{v}\frac{s/(2\pi f_{1})+1}{s/(2\pi f_{v})+1},w_{u}(s)=\frac{z_{u}}{i_{c}}=K_{u}\frac{s/(2\pi f_{1})+1}{s/(2\pi f_{u})+1}$$

where *f*_1_, *f_v_*, and *f_u_* are angular frequencies. *K_v_* and *K_u_* are constant gains. With the aim of obtaining the augmented system matrix, the performance variables *W_v_*(*s*) and *W_u_*(*s*) are expressed in the state-space form of Equation (15).

(15) $$\begin{array}{ccc} \begin{array}{c} {\dot{x}}_{v}=A_{v}x_{v}+B_{v}z_{s}\\ z_{v}=C_{v}x_{v}+D_{v}z_{s} \end{array} & , & \begin{array}{c} {\dot{x}}_{u}=A_{u}x_{u}+B_{u}i_{c}\\ z_{u}=C_{u}x_{u}+Dui_{c} \end{array} \end{array}$$

By substituting Equation (15) into Equation (13), the augmented system Equation (16) is obtained. The final augmented system will be used in the synthesis of the time-delay output feedback chatter control method.

(16) $$\begin{array}{l} \dot{\eta }(t)=\hat{A_{l}}\eta (t)+\hat{A_{l\tau }}\eta (t-\tau )+\hat{B_{li}}i_{c}(t)+\hat{B_{ld}}d(t)\\ z(t)=\hat{C_{l1}}\eta (t)+\hat{D_{l}}i_{c}(t)\\ z_{s}(t)=\hat{C_{l2}}\eta (t) \end{array}$$

Measured displacement *Z_s_* at AMB is used as the feedback, and the time-delay feedback control law is set as follows:

(17) $$\begin{array}{l} \dot{\varepsilon }\left(t\right)=A\varepsilon \left(t\right)+A_{\tau }\varepsilon \left(t-\tau \right)+Bz_{s}\left(t\right)+B_{\tau }z_{s}\left(t-\tau \right)\\ i_{c}(t)=\mathbb{C}\varepsilon (t)+{\mathbb{C}}_{\tau }\varepsilon (t-\tau )+Dz_{s}(t)+D_{\tau }z_{s}(t-\tau ) \end{array}$$

where $\varepsilon \in R^{k}$ is the state of the controller. A, $A_{\tau }$, B, $B_{\tau }$, C, $C_{\tau }$, D, $D_{\tau }$ are undetermined real constant matrices with appropriate dimensions. When the controller represented by Equation (17) is applied to the augmented system described in Equation (16), the closed-loop augmented system in Equation (18) can be acquired.

(18) $$\begin{array}{l} \dot{X}(t)=\hat{A}X(t)+\hat{A_{\tau }}X(t-\tau )+\hat{B_{d}}d(t)\\ Z(t)=\hat{C}X(t)+\hat{C_{\tau }}X(t-\tau )\\ i_{c}(t)=\Gamma X(t)+\Gamma_{t}X(t-\tau ) \end{array}$$

where $\hat{A}=\left[\begin{array}{cc} \hat{A_{l}}+\hat{B_{li}}D\hat{C_{l2}} & \hat{B_{li}}C\\ B\hat{C_{l2}} & A \end{array}\right]$, $\hat{A_{\tau }}=\left[\begin{array}{cc} \hat{A_{l\tau }}{+\hat{B_{li}}D}_{\tau }\hat{C_{l2}} & {\hat{B_{li}}C}_{\tau }\\ B_{\tau }\hat{C_{l2}} & A_{\tau } \end{array}\right]$, $\hat{B_{d}}=\left[\begin{array}{c} \hat{B_{ld}}\\ 0 \end{array}\right]$, $\hat{C_{\tau }}=\left[\begin{array}{cc} {\hat{D_{l}}D}_{\tau }\hat{C_{l2}} & \hat{D_{l}}C_{\tau } \end{array}\right]$, $\Gamma =\left[\begin{array}{cc} D\hat{C_{l2}} & C \end{array}\right]$, $\Gamma_{\tau }=\left[\begin{array}{cc} D_{\tau }\hat{C_{l2}} & C_{\tau } \end{array}\right]$.

### 3.2. Synthesis Problem

In order to derive the LMIs for this system, a Lyapunov function with the integral quadratic form is introduced.

(19) $$V(X,t)=X^{T}(t)PX(t)+\int_{t-\tau }^{t}X^{T}(\alpha )QX(\alpha )d\alpha$$

where *P* and *Q* are symmetric positive definite matrices. If the following conditions hold, the system is stable, and the H-infinity norm of the closed-loop transfer function from the input *d*(*t*) to the control output *Z*(*t*) of the system is less than the constant *γ*.

(20) $$\dot{V}(X,t)+Z^{T}(t)Z(t)-\gamma^{2}d^{T}(t)d(t)<0$$

Substituting *Z*(*t*) in Equation (18) and Lyapunov function Equation (19) into inequation (20), the following matrix inequation (21) can be obtained after calculating the solution:

(21) $${\left[\begin{array}{c} X\left(t\right)\\ X\left(t-\tau \right)\\ d\left(t\right) \end{array}\right]}^{T}\left[\begin{array}{ccc} P\hat{A}+{\hat{A}}^{T}P+Q+{\hat{C}}^{T}\hat{C} & P\hat{A_{\tau }}+{\hat{C}}^{T}\hat{C_{\tau }} & P\hat{B_{d}}\\ {\hat{A}}_{\tau }^{T}P+{\hat{C_{\tau }}}^{T}\hat{C} & -Q+{\hat{C_{\tau }}}^{T}\hat{C_{\tau }} & 0\\ {\hat{B_{d}}}^{T}P & 0 & -\gamma^{2}I \end{array}\right]\left[\begin{array}{c} X\left(t\right)\\ X\left(t-\tau \right)\\ d\left(t\right) \end{array}\right]<0$$

To obtain the equivalent matrix inequation, the Schur complements are applied. Since *P* serves as a symmetric positive definite matrix, in order to obtain the LMI inequation, *P* is defined as follows, where *M* and *N* are both symmetric matrices of dimension *n* × *n*:

(22) $$P=\left[\begin{array}{cc} U & M\\ M^{T} & \Omega \end{array}\right],P^{-1}=\left[\begin{array}{cc} V & N\\ N^{T} & \Theta \end{array}\right]$$

Define $\Delta_{1}=\left[\begin{array}{cc} V & I\\ N^{T} & 0 \end{array}\right]$, $\Delta_{2}=\left[\begin{array}{cc} I & U\\ 0 & M^{T} \end{array}\right]$, $P\Delta_{1}=\Delta_{2}$, ${Q{=\Delta }_{1}}^{T}Q\Delta_{1}=\left[\begin{array}{cc} Q_{11} & Q_{12}\\ Q_{21} & Q_{22} \end{array}\right]$. Given positive definite matrices V, U and full-rank matrices M, N, multiply the left side of inequation (21) by diag{Δ_1_^T^, Δ_1_^T^, I, I} and the right side by diag{Δ_1_, Δ_1_, I, I}. We simplify the matrix by defining the following variable substitution formula **A**, **Aτ**, **B**, **Bτ**, **C**, **Cτ**, **D**, **Dτ** to obtain inequation (23).

(23) $$\Psi =\left[\begin{array}{cccccc} \Psi_{11} & \Psi_{12} & \hat{A_{l\tau }}V+\hat{B_{li}}C & \hat{A_{l\tau }}+\hat{B_{li}}D_{\tau }\hat{C_{l2}} & \hat{B_{ld}} & V{\hat{C_{l1}}}^{T}+C^{T}{\hat{D_{l}}}^{T}\\ \Psi_{21} & \Psi_{22} & A\tau & U^{T}\hat{A_{l\tau }}+B_{\tau }\hat{C_{l2}} & U\hat{B_{ld}} & {\hat{C_{l1}}}^{T}+{\hat{C_{l2}}}^{T}D^{T}{\hat{D_{l}}}^{T}\\ {}* & * & -Q_{11} & -Q_{12} & 0 & {C_{\tau }}^{T}{\hat{D_{l}}}^{T}\\ {}* & * & -Q_{21} & -Q_{22} & 0 & {\hat{C_{l2}}}^{T}{\hat{D_{l}}}^{T}{D_{\tau }}^{T}\\ {}* & * & * & * & -\gamma^{2}I & 0\\ {}* & * & * & * & 0 & -I \end{array}\right]<0$$

where

$$\begin{array}{l} \Psi_{11}=\hat{A_{l}}V+V{\hat{A_{l}}}^{T}+\hat{B_{li}}C+C^{T}{\hat{B_{li}}}^{T}+Q_{11},\Psi_{12}=\hat{A_{l}}+\hat{B_{li}}D\hat{C_{l2}}+A^{T}+Q_{12}\\ \Psi_{21}=A+{\hat{A_{l}}}^{T}+{\hat{C_{l2}}}^{T}D^{T}{\hat{B_{li}}}^{T}+Q_{21},\Psi_{22}=U^{T}\hat{A_{l}}+B\hat{C_{l2}}+{\hat{A_{l}}}^{T}U^{T}+{\hat{C_{l2}}}^{T}B^{T}+Q_{22} \end{array}$$

Due to the output current constraints of the actuator in practical physical applications, the control inputs should satisfy the following hard constraints:

(24) $$\begin{array}{l} i_{c}(t)=\Gamma X(t)+\Gamma_{t}X(t-\tau )\\ \left|i_{c}(t)\right|\leq i_{cmax} \end{array}$$

where ${i_{c}}_{max}$ is a given definite constant. According to inequation (20), as well as the Schur complement lemma, eventually, we obtain inequation (25).

(25) $$\Theta =\left[\begin{array}{ccccc} -I & \sqrt{2\rho }C & \sqrt{2\rho }D\hat{C_{l2}} & \sqrt{2\rho }C_{\tau } & \sqrt{2\rho }D_{\tau }\hat{C_{l2}}\\ {}* & -i_{cmax}^{2}V & -i_{cmax}^{2}I & 0 & 0\\ {}* & -i_{cmax}^{2}I & -i_{cmax}^{2}U & 0 & 0\\ {}* & * & * & -i_{cmax}^{2}V & -i_{cmax}^{2}I\\ {}* & * & * & -i_{cmax}^{2}I & -i_{cmax}^{2}U \end{array}\right]<0$$

The controller is acquired through a numerical solution to the following LMI feasibility problem:

Given $\gamma ,\rho {{,i}_{c}}_{max},\tau$,

find V = V^T^,U = U^T^, Q = Q^T^, A, Aτ, **B**, **Bτ**, **C**, **Cτ**, **D**, **Dτ**

s.t Φ<0, Θ<0, $\left[\begin{array}{cc} V & I\\ I & U \end{array}\right]>0$, $\left[\begin{array}{cc} Q_{11} & Q_{12}\\ Q_{21} & Q_{22} \end{array}\right]>0$.

According to *MN^T^ = I* − *V* in Equation (22), after solving the above inequation to obtain *U* and *V*, the full-rank matrices *U* and *V* are obtained by singular value decomposition of matrix *I* − *V*. Then, the required parameters of the controller are obtained according to the variable substitution formula.

(26) $$\begin{array}{l} A=M^{-1}(A-(U^{T}(\hat{A_{l}}+\hat{B_{li}}D\hat{C_{l2}})+MB\hat{C_{l2}})V-U^{T}\hat{B_{li}}\mathbb{C}N^{T})N^{-T}\\ A_{\tau }=M^{-1}(A_{\tau }-(U^{T}(\hat{A_{l\tau }}+\hat{B_{li}}D_{\tau }\hat{C_{l2}})+MB_{\tau }\hat{C_{l2}})V-U^{T}\hat{B_{li}}{\mathbb{C}}_{\tau }N^{T})N^{-T}\\ B=M^{-1}(B-U^{T}\hat{B_{li}}D),B_{\tau }=M^{-1}(B_{\tau }-U^{T}\hat{B_{li}}D_{\tau })\\ \mathbb{C}=(C-D\hat{C_{l2}}V)N^{-T},{\mathbb{C}}_{\tau }=(C_{\tau }-D_{\tau }\hat{C_{l2}}V)N^{-T}\\ D=D,D_{\tau }=D_{\tau } \end{array}$$

## 4. Simulation Results

In this section, the output feedback chatter controller that has been proposed is adapted to the coupled system in Section 2. To verify the effectiveness of the proposed method, a demonstrative example of low immersion milling is provided.

**Figure 6:**
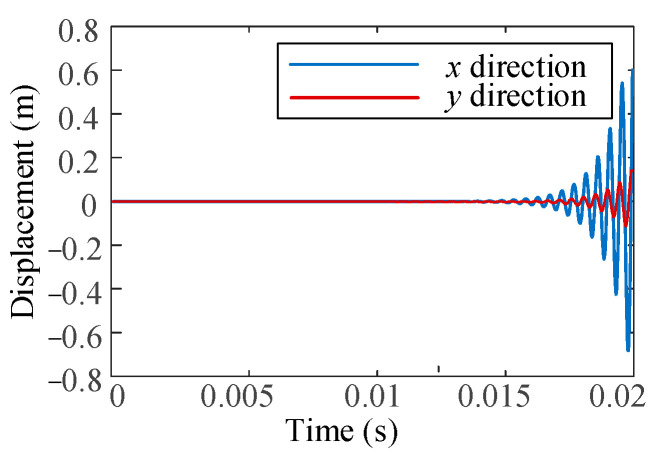
Displacement response without control.

The model parameters utilized in the simulation are from reference [25]. When no active control force is exerted, the displacement output response of the open-loop system is depicted in Figure 6. It indicates that the milling vibration will gradually intensify in the absence of additional control forces.

To validate whether the proposed control method is effective in both the full immersion milling conditions and the low immersion milling conditions, numerical simulations are carried out under milling conditions C1 and C2 shown in Table 2. C1 corresponds to the cutting parameters in full immersion milling, while C2 is the milling parameters in low immersion milling. The proposed control method is compared with the H-infinity controller in [41]. Intending to demonstrate the superiority of the proposed control method while considering the current limit, we divided the simulation for each cutting condition into two categories: one that considers the current constraints, and another that does not. We set the maximum output control current to 6 A in the simulation.

**Figure 7:**
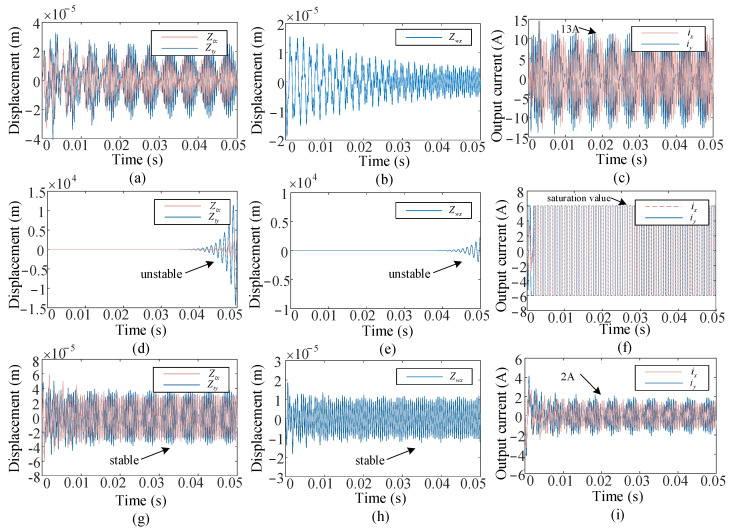
Simulation results in milling conditions C1: (**a**–**c**) Displacement of the tooltip, displacement of workpiece, and output current under H-infinity controller. (**d**–**f**) Displacement of the tooltip, displacement of workpiece, and output current under H-infinity controller with current constraints. (**g**–**i**) Displacement of the tooltip, displacement of workpiece, and output current under the proposed controller.

In fully immersed milling conditions C1, we compared the proposed control method with the H-infinity controller. The simulation results are illustrated in Figure 7. It can be seen from Figure 7a–c,g–i that when the current constraints are not considered, both control methods demonstrate comparable effectiveness. The displacement of the tooltip eventually stabilized at 2.5 × 10^−5^ m, and the displacement of the workpiece eventually stabilized at 1 × 10^−5^ m. At this point, the currents required by the two controllers are 10 A and 3 A, respectively. Since the H-infinity controller does not take the physical limitations into account, the energy required exceeds the maximum current specified that the actuator can provide to achieve the same control effect. While current constraints are taken into account, the results vary significantly. Simulation results for the H-infinity controller are presented in Figure 7d–f. When the control current reaches saturation, the H-infinity controller becomes ineffective, leading to a gradual loss of system stability and the immediate onset of chatter phenomena. The proposed controller incorporates output current constraints ensuring the required current is merely 3 A, which is consistently maintained within the allowable range, resulting in greater energy savings compared with the H-infinity controller.

**Figure 8:**
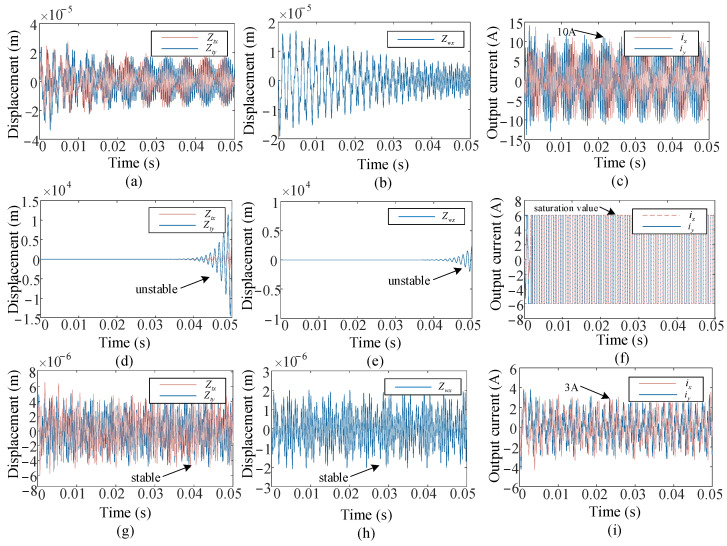
Simulation results in milling conditions C2: (**a**–**c**) Displacement of the tooltip, displacement of workpiece, and output current under H-infinity controller. (**d**–**f**) Displacement of the tooltip, displacement of workpiece, and output current under H-infinity controller with current constraints. (**g**–**i**) Displacement of the tooltip, displacement of workpiece, and output current under the proposed controller.

We conducted numerical simulations under low immersion milling conditions C2 and compared the proposed control method with the H-infinity controller as well. Nevertheless, unlike the results observed under full immersion milling conditions C1, the two control methods exhibited some differences. As shown in Figure 8, although both systems eventually stabilize under low immersion milling conditions, the displacement response and needed output current achieved with the proposed control method are much smaller than compared with those achieved with the H-infinity controller. The displacement response of the tooltip and workpiece with the H-infinity controller ultimately stabilized at 2 × 10^−5^ m and 1 × 10^−5^ m, respectively, while the required current still exceeds the maximum current that the actuator can withstand. When the current is strictly limited to 6 A, the system becomes unstable. Nevertheless, the simulated displacement of the tooltip and workpiece with the proposed controller ultimately converges to 6 × 10^−6^ m and 2 × 10^−6^ m, while the control current still remains within the specified limits. This implies that the proposed controller demonstrates superior performance in low immersion milling conditions.

**Figure 9:**
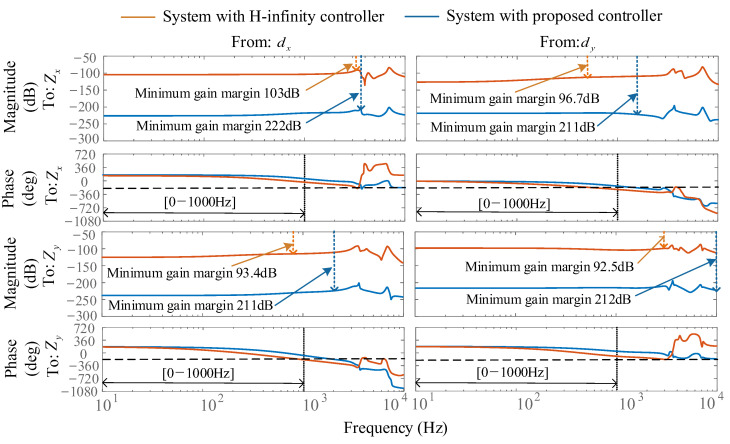
Bode plot.

In the controller design process outlined in Section 3, we also take into account the bandwidth limitations of the controller. To assess the impact of integrating weighting functions into the controller synthesis problem, we conducted simulation validation. In this simulation, the controller bandwidth is restricted to 1000 Hz. The constant gains *K_v_* and *K_u_* are selected based on the actual machining parameters. Taking low immersion milling conditions C2 as an example, Figure 9 compares the Bode plots of two systems, a system with the H-infinity controller and a system with the proposed controller, illustrating the frequency response characteristics of the outputs of the *Z_x-_* and *Z_y_*-controlled system when reacting to the input disturbances *d_x_* and *d_y_*. In the lower-left group of figures, the minimum gain margin of the system with the H-infinity controller is 93.4 dB, with a phase crossover frequency of 750 Hz. When employing a frequency-dependent weighting function in the system with the proposed controller, the minimum gain margin increases to 211 dB, and the phase crossover at −180° shifts to 1200 Hz, significantly enhancing the gain margin of the closed-loop system. This improvement is consistently observed in the other groups of Figure 9, indicating that the proposed controller provides superior stability to the system.

In summary, the above simulation results illustrate that the controller considering the thin-walled workpiece and the weak rigidity of the tool is capable of effectively suppressing the vibrations of the workpieces and tools under both full immersion and low immersion milling conditions. Simultaneously, the additional physical constraints considered by the controller enable the control force to save more energy.

## 5. Experimental Results and Discussions

**Figure 10:**
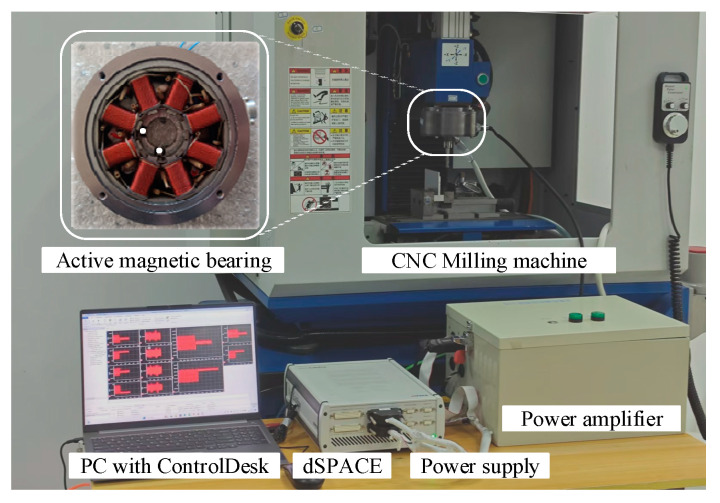
Active control structure.

In this section, experiments are conducted on a CNC milling machine as exposed in Figure 10. The active tool holder structure designed in Section 2 with an AMB as the actuator is attached at the terminal part of the milling machine’s spindle. To obtain the parameters required for the experiments, parameter identification is carried out first, and finally, the proposed active control method is verified under low immersion milling conditions.

### 5.1. System Identification

Figure 10 physically presents the designed active control structure. Eddy current displacement sensors are placed at the terminal part of the spindle in the *x* and *y* directions to measure vibration in the position of the tool holder. In the active control process, the displacement sensor measures the vibration and feeds the measurement signal back to the dSPACE MicroLabBox RT-1202 (dSPCE, Paderborn, Germany), which generates the required control signal. The power amplifier possessing a gain of 1 A/V, converts the necessary control signal into control current. Then, the current is directly outputted to the active magnetic bearing, thus realizing vibration control.

**Figure 11:**
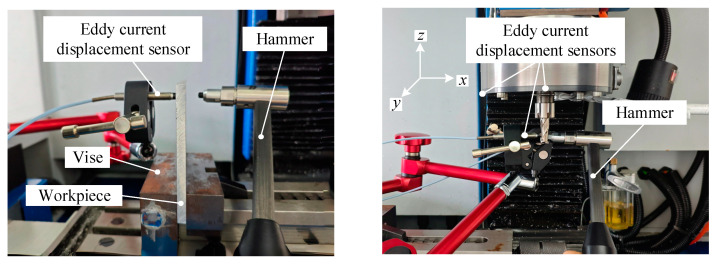
Experimental setup for parameter identification.

On this basis, the hammer test, as well as the sweep frequency test, are carried out. Figure 11 shows the experimental setup for modal parameter identification. Eddy current displacement sensors (ZA-210500, Zhonghang Technology, Zhuzhou, China) are installed in the *y* direction regarding the workpiece and in the *x* and *y* with respect to the tooltip and tool holder. The excitation is performed using an impact hammer. The material of the workpiece utilized in the experiment is Al6061, size 90 mm × 90 mm × 6 mm. The milling tool utilized in this experiment is an end milling cutter, and its specifications are shown in Table 3.

**Figure 12:**
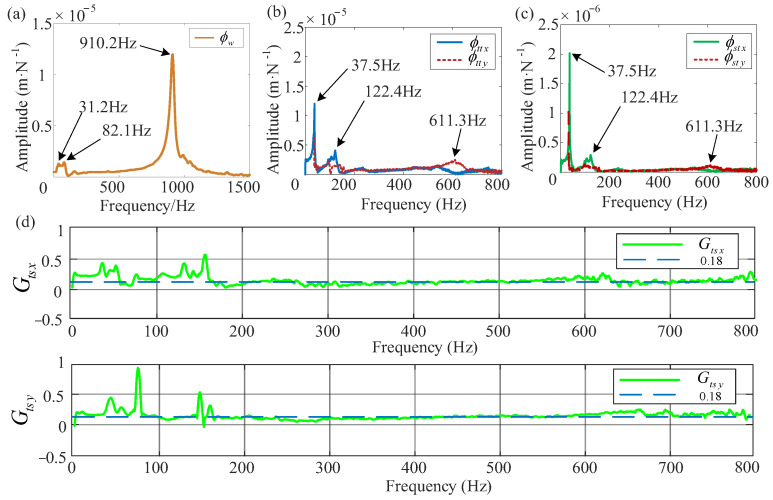
Measured frequency response functions: (**a**) response of the workpiece when hammer is excited, (**b**) response of the tooltip when hammer is excited, (**c**) response of the tool holder when hammer is excited, (**d**) transfer function of displacement response between the tooltip and the tool holder.

Since the workpiece’s stiffness in the *y* direction is significantly greater compared with that in the *x* direction. The workpiece is deemed rigid in the *y* direction, with consideration being given solely to the dynamic characteristics in the *x* direction. In order to measure the frequency response function *ϕ_w_*(*s*), a hammer excitation is applied to the workpiece. The displacement response of the workpiece is obtained by an eddy current displacement sensor, and the frequency response function derived from data processing is illustrated in Figure 12a. Figure 12b depicts the frequency response function *ϕ_tt_*(*s*), which represents the response of the tooltip upon hammer excitation. This function is acquired by applying impacts to the tooltip from the *x* and *y* directions with a hammer. Additionally, *ϕ_st_*(*s*) represents the transfer function between the tool holder and the hammer excitation, as shown in Figure 12c. When excitation is exerted at the tooltip position, displacement sensors installed at both the tool holder and tooltip positions simultaneously measure the corresponding responses. For the purpose of streamlining the design of the controller in the subsequent section, it is assumed that the transfer functions *ϕ_st_*(*s*)/*ϕ_tt_*(*s*) in both the *x* and *y* directions take on a constant value with a linear relationship. The responses in the *x* and *y* directions are ultimately represented as the transfer function depicted in Figure 12d. This transfer function can be approximated as a constant value of 0.18. Considering that the designed controller has strong robustness, the deviation between its approximation and the experimental results can also be considered as a disturbance to the system. 

### 5.2. Formatting of Mathematical Components

Experimental verification is carried out on the CNC milling machine exposed in Figure 10. In the experiments, the displacement signal is measured by eddy current displacement sensors with a sampling frequency of 10 kHz. The displacement measurement signal at the tool holder is used as feedback for the system. The spindle speed in experiments is 3000 rpm. Continuous milling experiments are carried out under low immersion conditions with an axial depth of cut equal to 5 mm and a radial depth of cut amounting to 3 mm. The control effect of the controller designed in this paper is compared with that of the H-infinity controller when there is a current constraint. A bias current of 2 A and a maximum control current of 0.75 A are set.

**Figure 13:**
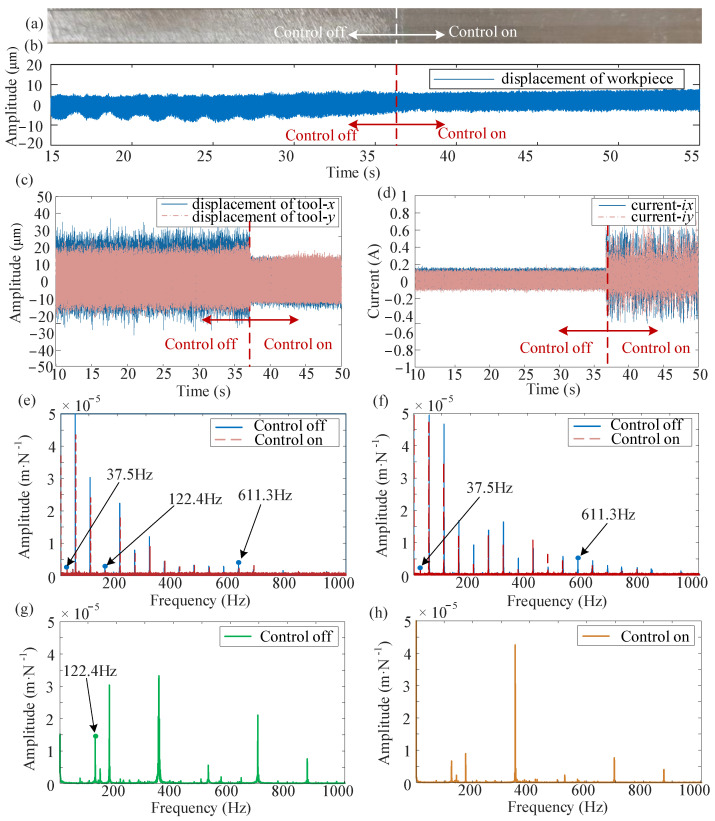
Control effect of the proposed controller: (**a**) workpiece surface, (**b**) workpiece displacement response, (**c**) tool displacement response, (**d**) control current, (**e**) frequency spectra of the tool before and after control in the *x* direction, (**f**) frequency spectra of the tool before and after control in the *y* direction, (**g**,**h**) frequency spectra of workpiece vibration signal before and after control.

The results of the milling experiments are presented in Figure 13. It covers both the situations when the proposed controller is applied and when it is not, while taking current constraints into account. The designed controller activates at the 37th second. As illustrated in Figure 13a, the surface of the workpiece becomes smoother following the implementation of active control. Figure 13b,c display the measured displacements of the workpiece and the tool during the milling process, respectively. When the controller is operational, the tool displacement converged from 40 μm to a steady-state displacement of 25 μm, while the workpiece displacement decreased from 60 μm to 40 μm, resulting in a 30% reduction in vibration. The actual required control current remains consistently below 0.75 A. Furthermore, the spectrogram confirms the effective reduction in amplitude around the chattering frequency. The spectrogram of the workpiece before and after control is shown in Figure 13g,h, while the spectrograms of the tool are presented in Figure 13e,f. From the experiment results in Figure 12, we obtained the main frequencies of chatter. The primary chattering component of the workpiece (31.2 Hz, 82.1 Hz, and 910.2 Hz) and the main chattering components of the tool (37.5 Hz, 122.4 Hz, and 611.3 Hz). It is obviously observed from Figure 13 that the chatter frequencies of 37.5 Hz, 122.4 Hz, and 611.3 Hz appeared in the spectrogram of the tool displacement before the chatter control was performed, and the chatter frequency of 122.4 Hz appeared in the workpiece, while after the control, the chatter frequency disappeared. Only the rotational frequency, the over-tooth frequency, and its harmonics remain in the spectrogram. This indicates that the developed controller effectively suppresses chatter vibrations.

**Figure 14:**
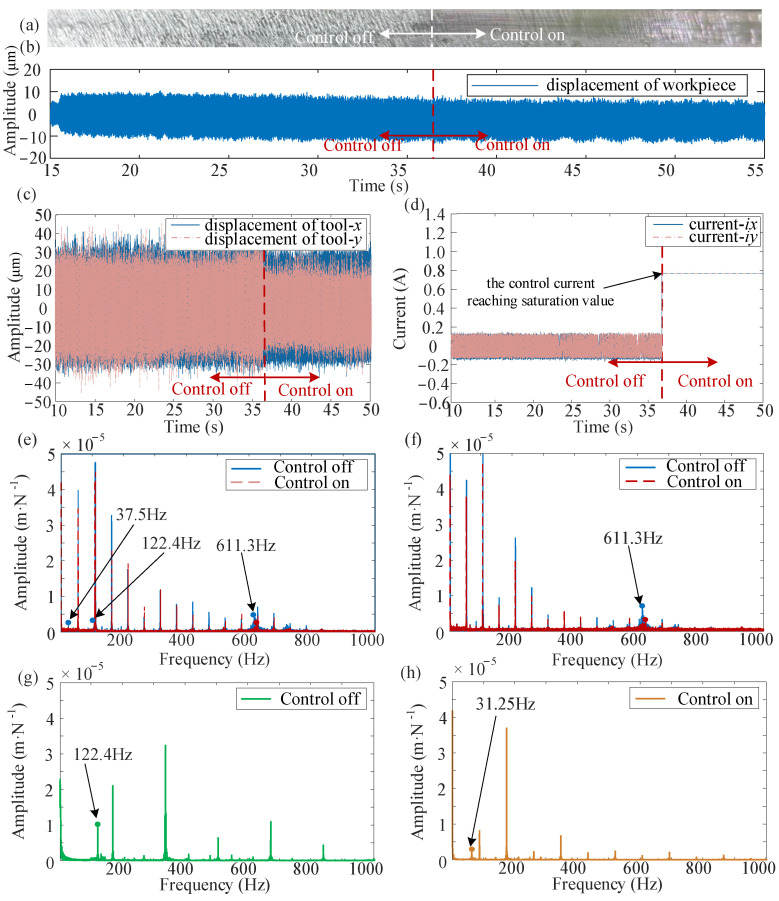
Control effect of H-infinity controller: (**a**) workpiece surface, (**b**) workpiece displacement response, (**c**) tool displacement response, (**d**) control current, (**e**) frequency spectra of the tool before and after control in the x direction, (**f**) frequency spectra of the tool before and after control in the y direction, (**g**,**h**) frequency spectra of workpiece vibration signal before and after control.

The experimental results with control off and control on while the H-infinity controller is employed are illustrated in Figure 14. The controller is activated at the 37th second, and the control current becomes saturated due to the upper current limit setting of 0.75 A. Consequently, the control performance under the H-infinity controller is inferior compared with the proposed controller. The chatter frequencies of 37.5 Hz, 122.4 Hz, and 611.3 Hz appeared in the spectrogram of the tool displacement before the chatter control was performed, and the chatter frequency of 122.4 Hz appeared in the workpiece, while after the control, the chatter frequency present still. The displacements of both the tool and the workpiece are reduced by only 10%, and the convergence effect for both the workpiece and the tool is significantly lower than that of the proposed controller. This indicates that the proposed active control system provides superior suppression of chattering.

## 6. Conclusions

In this work, an active electromagnetic tool holder system is proposed for milling chatter of thin-walled workpieces under low immersion milling conditions. The main contributions and conclusions are as follows:

1. The active electromagnetic tool holder system consists of an active structure with an active magnetic bearing mounted at the tool holder position and a time-delay output feedback chatter control method designed for milling conditions with low immersion ratios. The proposed structure allows the generation of a noncontact force to be employed directly to the tool holder and closer to the milling point.
2. To address the issue of an unstable cutting process caused by thin-walled workpieces and weak tool rigidity under low immersion machining conditions, the coupling effect between the tool and the workpiece is taken into account, along with the dynamic characteristics of both during the controller design.
3. The actual physical limitations, including the bandwidth of the power amplifier and the output current of the actuator, are also taken into account during the controller design process. The implementation of a frequency-dependent weighting function ensures the superior stability of the controlled system within the set range and conserves control energy. In addition, current saturation can be effectively prevented by current limitation to a certain range.
4. Simulations and experiments of milling under low immersion conditions demonstrate that the proposed active electromagnetic tool holder system has a positive effect on reducing the vibration of both thin-walled workpieces and tools.

## Figures and Tables

**Table 1:** Designed electromagnetic actuator parameters.

Parameter	Value
Air gap, $c_{0}$	0.5 mm
Number of coil turns for two neighboring poles, N	81
The angular value between two adjacent poles, 2α	45°
Bias current, $i_{0}$	2 A
Maximum control current, ${i_{c}}_{max}$	8 A
Maximum control force, $F_{a}$	180 N

**Table 2:** Parameters of milling conditions A and B.

Milling Conditions	Speed/r·min^−1^	Radial Immersion Ratio	Axial Depth of Cut/mm	Milling Type
C1	30,000	1	1	Up milling
C2	15,000	0.3	2	Up milling

**Table 3:** Specifications of the milling tool in the experiment.

Tooth Number	Diameter /mm	Overhang /mm	Helix Angle/◦	Rake and Flank Angle/◦	Runout Value/mm
2	10	48	45	16	0.01

## Data Availability

Data are contained within the article.

## Nomenclature

*A_n_*	The magnetic circuit’s cross-sectional area, [m^2^]
*AMB*	Active magnetic bearing
*D*	The diameter of the milling tool, [m]
*F_a_*	The control force imposed by actuator, [N]
*F_s_*_,_ *F_d_*, *F_t_,*	The static, dynamic, total milling force, [N]
*F_tj_*, *F_rj_*	The tangential, radial cutting force, [N]
*G_w_*(*s*)	The transfer function from the milling force to the workpiece
*G_st_*(*s*)	The transfer function from the milling force to the measured position of the spindle system
*G_sa_*(*s*)	The transfer function of the controlled force generated by the actuator to the measured position
*G_ts_*(*s*)	The transfer function from the measured position to the tooltip
*H*(*t*), *H*_0_(*t*)	The milling force coefficient matrixes
*K_r_*, *K_t_*	The radial and tangential milling force coefficients, [Pa]
*N*	Tooth number
*Z_t_*, *Z_w_*	The displacement at the tooltip, workpiece, [m]
*Z_s_*	The displacement signal of the actuator position on the tool holder, [m]
*Ω*	The spindle speed, [r·min^−1^]
*τ*	Tooth passing period, [s]
*ϕ_w_*(*s*), *ϕ_tt_*(*s*)	The frequency response function from the milling force to the workpiece, tooltip
*ϕ_st_*(*s*)	The transfer function between the tool holder and the hammer excitation
*i_c_*_,_ *i_0_*	The control, bias current, [A]
*μ*_0_	The vacuum permeability, [V·s/(A·m)]
*c*_0_	The air gap, [m]
*k_i_* , *k_s_*	The current, displacement stiffness coefficient, [N/A, m/A]
*h_s_*, *h_d_, h*(*ϕ_j_*)	The static, cutting, dynamic, total thickness, [m]
*f_z_*	The feed per tooth, [m]
*ϕ_j_*	The instantaneous angular position of tooth *j*, [rad]
*b*	The axial cutting depth, [m]
*ϕ_en,_ ϕ_ex_*	The starting and exiting immersion angles between the tool and the entry point, [rad]
*a*	The radial cutting depth, [m]
*f_1_*, *f_v_*, *f_u_*	The angular frequencies
